# What can be learned from lecturers’ knowledge and self-efficacy for online teaching during the Covid-19 pandemic to promote online teaching in higher education

**DOI:** 10.1371/journal.pone.0275459

**Published:** 2022-10-05

**Authors:** Ron Blonder, Yael Feldman-Maggor, Shelley Rap

**Affiliations:** Department of Science Teaching, Weizmann Institute of Science, Rehovot, Israel; The Education University of Hong Kong, HONG KONG

## Abstract

The experience of graduate degree lecturers in the natural sciences when they switched to online teaching during the Covid-19 pandemic is described. The shift to online teaching throughout the pandemic provided an opportunity to evaluate how lecturers integrate technology into their teaching and what they need to improve their remote teaching. This study used a twofold perspective of TPACK (Technological Pedagogical Content Knowledge) and self-efficacy in online education. Its data were derived from pre-and post-questionnaires, comprising closed and open-ended questions, given at the start and end of the semester. We found that lecturers focused on learning and applying technological and techno-pedagogical knowledge but paid less attention to the integration of three components: technology, pedagogy, and scientific content. Although no statistically significant differences in lecturers’ perceived self-efficacy was found between the start and the end of the semester, at the end of the semester we found a statistically significant correlation between the variables involved in building self-efficacy in online teaching: (1) satisfaction with online teaching and the belief that (2) technology promotes teaching, student interactions, participation, and engagement. Our results enabled us to identify the knowledge aspects that lecturers implemented initiatively and to better understand what aspects required more professional development training. In addition, the results emphasized the importance of developing the lecturers’ self-efficacy for online teaching. These insights can help to improve and enhance online teaching in higher education.

## Introduction

Integrating technology into higher education teaching and learning requires changing both the lecturers’ knowledge of educational technology and their beliefs, stands, and pedagogical perceptions [1, 2]. In the past few years, universities worldwide have significantly expanded distance learning, using integrated video-based technological means, such as online homework lectures and lessons that complement traditional classes [3]. Numerous universities have been offering whole courses that are open to the public on the internet [4]. Although online learning and its recognition have been discussed in Israel as well [5], frontal instruction was the most widespread mode of academic teaching before the Covid-19 outbreak. Most lecturers adhered to the traditional lecture mode, using technology only to support frontal teaching [6]. However, the Covid-19 outbreak caused all the higher education lecturers to switch to online teaching immediately and without preparation [7]. These enable us to examine those lecturers’ beliefs and knowledge when they switched to online teaching with the Covid-19 outbreak. No less important is revealing how practical experience using technological tools in teaching affected the lecturers’ knowledge and beliefs.

To this end, we chose two perspectives: one refers to the lecturers’ knowledge and the other to their beliefs regarding online teaching. Both perspectives (knowledge and beliefs) represent essential components of quality online teaching [8]. TPACK (Technological pedagogical content knowledge) [9] is used to address the necessary knowledge to integrate technology into teaching, and self-efficacy in online teaching [10] is used to address the lecturers’ beliefs regarding their ability to teach online.

TPACK is based on the Shulman’s theoretical framework of teachers’ pedagogical content knowledge (PCK) [11]. It expands PCK by characterizing the types of knowledge required to promote effective integration of technology into teaching while considering content and pedagogy [12]. The TPACK framework proposes seven distinct categories of teacher knowledge. In addition to the three categories suggested by Shulman [11, 13], the TPACK framework includes four more categories related to technology: Technological Knowledge (TK) represents knowledge on how to use technologies (e.g., how to operate computer programs and different technological applications). Technological Pedagogical Knowledge (TPK) is an extension of general pedagogical knowledge (PK). It represents the integration of technology with general pedagogical strategies (e.g., how to engage students in technology-oriented activities and evaluate online learning). Teachers who possess this kind of knowledge understand the impact of technology on general pedagogical practices that are not content-specific. Technological Content Knowledge (TCK) represents knowledge of technological tools that practitioners use within a specific discipline. In science education, it represents the technological knowledge that a scientist would have and that teachers want their students to acquire (e.g., use of databases by scientists [14]). Finally, TPACK represents the ability of teachers or lecturers to combine those three knowledge components (technology, pedagogy, and content) to advance their teaching objectives. TPACK is primarily achieved when a teacher knows how technological tools transform pedagogical strategies and content representations for teaching particular topics, and how technological tools in education and representations affect a student’s understanding of these topics (e.g., the use of visualization tools to support students’ understanding of scientific phenomena [15, 16]). Online teaching is a notable example of implementing TPACK in curricular teaching [17–19].

Studies have indicated that teachers who undergo training that includes TPACK tend to realize that this approach to implementing technology in teaching promotes teaching and contributes to the learning process. Teachers who undergo such training understand that content, pedagogy, and technology must be integrated rather than perceived as separate components in their teaching [20, 21].

TPACK is recognized as one of the leading theoretical frameworks for analyzing technology integration in teaching [22]. Most of the existing literature about TPACK has focused on school teachers [23]. Whereas, the literature on TPACK and higher education is far less developed. For example, in a research book on the topic of TPACK published in 2016 [24], only one chapter out of 20 was devoted to TPACK in higher education. Nevertheless, some important studies were conducted in the context of higher education [24–27]. These works focused on teacher education, specifically on the development of pre-service teacher TPACK [24], faculty development programs [25, 26], and course design [27]. In the current research, we aimed to expand the existing research on TPACK and higher education. Specifically, we studied the TPACK of lecturers who had to teach online when there were no other alternatives and did not receive appropriate professional training.

However, TPACK is insufficient when new technology is adopted. In a recent review dealing with the integration of computational chemistry in chemistry courses, Tuvi-Arad [28] presented several challenges that inhibit the integration of advanced technology. First, she mentioned the TK of the lecturers and that they should be familiar with the relevant software. However, in her thorough review, she found evidence that lecturers’ self-efficacy beliefs and attitudes can influence technology integration. This insight is supported in a previous review of research about TPACK [29], which concluded with a suggestion to conduct an interdisciplinary study of TPACK with other theoretical frameworks related to the study of technology integration. The current study was designed to address this aspect and investigate how the TPACK framework intersects with the self-efficacy construct derived from Bandura’s social cognitive theory [10].

To employ an innovative technological tool in teaching, lecturers must believe that they can use a particular tool and enhance the students’ learning [10]. A sense of self-efficacy indicates the extent to which one is confident of functioning under different conditions and achieving the desired results. This belief affects that person’s decisions, choices, perseverance, and the ability to cope with various tasks throughout life [10]. A sense of self-efficacy gradually develops in an ongoing learning process, whereby a person accumulates knowledge from numerous sources about their functioning in different areas [30]. Bandura lists four resources that contribute to developing self-efficacy: (1) Past experiences (mastery experience), (2) Observing models (vicarious experience), (3) Positive feedback from one’s surroundings (verbal persuasion), and (4) Physical and psychological sensations and reactions (physiological state). Together, these four resources affect one’s perceived self-efficacy and the ability to realize one’s skills [10, 31]. According to Skinner (1996) [32], when self-efficacy is defined as an individual’s conviction that he or she can produce a controlling response, then high self-efficacy also implies the belief that a controlling response (a means–ends connection) already exists. However, in the current research, we follow a relevant definition of self-efficacy in the context of teaching that was provided by Gibson and Dembo (1984) [33]. The authors emphasized two components of teaching self-efficacy beliefs. The first is the belief that effective teaching can influence learning (outcome expectancy beliefs), and the second is having confidence in one’s own teaching abilities (self-efficacy beliefs). Having these two components should facilitate teachers to have a longer persistence, provide a greater focus in the classroom, and allow the teachers to exhibit different types of feedback, compared with teachers who have lower expectations concerning their ability to influence student learning [33].

This definition by Gibson and Dembo (1984) was adopted by other educational researchers [33] in science education research [34], and it is also applicable for science teaching using technology [35]. A strong sense of self-efficacy in using a technological tool is a vital precondition for lecturers to apply that tool in teaching [36]. Teachers with a high self-efficacy perception are more open to new ideas and are more willing to experience new methods and offer students new and unique learning opportunities and experiences [37]. To help lecturers develop this confidence, it is necessary to help them accumulating mastery experiences, engaging in vicarious experiences as well as persuasion that are likely to boost teachers’ self-efficacy [35, 38, 39].

As already mentioned, developing self-efficacy in using technology for teaching is a gradual, ongoing process [40]. However, the Covid-19 outbreak created circumstances in which higher education lecturers faced the need to implement new technological capabilities [41] instantly. The Weizmann Institute, for example, postponed the opening of the second semester by a week, and the lecturers underwent a short training course in the use of Zoom (https://zoom/us) to enable online teaching, which was similar to many other academic institutions worldwide [42]. These efforts enabled lecturers to better prepare for online teaching in the second semester of 2020, which was conducted entirely online using technological tools. Unlike previous research that studied lecturers who had switched to distance teaching, in the present study, the lecturers could not choose whether they wanted to switch to online instruction, since the Covid-19 crisis forced all of them to initiate online teaching [43]. A recent qualitative study, conducted in six universities in two countries, dealt with the challenges of online and e-learning systems. It identified five factors that influenced the successful use of an e-learning system during the Covid-19 pandemic [44]. The self-efficacy beliefs of lecturers and students were one of these factors, along with technological factors, trust, and cultural aspects. Therefore, there is a need to explore lecturers’ self-efficacy beliefs and knowledge while they are forced to shift to online teaching.

## Methodology

The study aimed to characterize the knowledge of science lecturers at the Weizmann Institute, explore their views about online teaching, and investigate the knowledge and self-efficacy they had developed over a semester-long course in online teaching.

### Research questions

Q1) What variables contribute to developing lecturers’ perceived self-efficacy in online teaching?

Q2) Which TPACK components did higher education science lecturers implement in online teaching?

This research compared the findings of pre-and post-questionnaires completed by Weizmann Institute of Science lecturers. The questionnaire’s development followed the theoretical TPACK and self-efficacy frameworks and included 14 multiple-choice questions on a rating scale, analyzed statistically, as well as open-ended questions that underwent qualitative analysis. The questionnaire was developed at the beginning of the pandemic (March 2020) and was validated using 197 high-school chemistry teachers [45]. These analyses enabled pre-post comparisons that examined how a semester of online teaching under Covid-19 circumstances affected the research variables and their correlation.

### The study population

Forty-six lecturers, who taught a course in the second semester of 2020, completed the preliminary (pre-) questionnaire, and 89 lecturers completed the post-questionnaire. The post-questionnaire was distributed in two cycles: the first cycle followed the second semester in 2020 and the second cycle followed the first semester in 2021 (each lecturer answered the post-questionnaire only once). The pre- and post-responses were not paired due to ethical restrictions. The lecturers all taught advanced degree courses offered to M.Sc. and Ph.D. natural science students at the Weizmann Institute. The lecturers’ teaching field, experience in online teaching, age, and characteristics regarding the courses are presented in Table 1.

**Table 1:** Demographic description of research participants: Remote online learning at the feinberg graduate school during the years 2020–2021.

Category	Sub-Category	Pre (N = 46)	Post (N = 89)
**Age**	Less than 30	4 (8.70%)	3 (3.37%)
	30–39	13 (28.26%)	14 (15.73%)
	40–49	12(26.09%)	23 (25.84%)
	50–60	11 (23.91%)	24 (26.97%)
	Over 60	6 (13.04%)	25 (28.09%)
**Role**	Lecturer	36 (78.26%)	81 (91.01%)
	Teaching Assistant	10 (21.74%)	8 (8.99%)
**Previous experience in remote teaching (before the Coronavirus outbreak)**	Yes	9 (19.57%)	10 (11.24%)
	No	37 (80.43%)	79 (88.76%)
**Discipline**	Chemistry	6 (13.04%)	12 (13.48%)
	Life Science	16 (34.78%)	27 (30.34%)
	Mathematics	2 (4.35%)	7 (7.87%)
	Physics	8 (17.39%)	13 (14.61%)
	Science Teaching	11 (23.92%)	20 (22.47%)
	Mixed Discipline	2 (4.35%)	6 (6.74%)
	Other	1 (2.17%)	4 (4.49%)
**Course Type**	Laboratory	NA	8 (8.99%)
	Lecture	NA	68 (76.40%)
	Seminar or Workshop	NA	13 (14.61%)

### Research tools

All the lecturers and teaching assistants, who taught in the second semester of 2020, received by email an anonymous research questionnaire that the Institute’s ethics committee had approved. The questionnaire was re-mailed to them in the last week of the semester. The data that support the findings of this study are available from the corresponding author upon reasonable request. The research received IRB (Institutional Review Board) approval from the Weizmann Institute of Science Ethics Committee and participants’ written consent was obtained.

The questionnaire consists of three main parts. The first part collected information about the lecturers’ background, including faculty, age, and previous experience in online teaching. The second part was an attitude questionnaire that comprised 14 questions about five variables (for original data see S5 Table in the Supporting Information section). Two of the five variables are components of self-efficacy in online teaching [35], presented in Table 2. The pre-questionnaire included four of the five categories. In developing the questionnaire, we applied a previous questionnaire developed to measure the views of chemistry teachers regarding online teaching during the Covid-19 period [45]. To validate the variables, we used factor analysis and a Cronbach’s alpha test (Table 2). In the third part, the respondents had to provide detailed answers to the following open questions:

- What efforts did you put into preparing and teaching the online course?
- Did you lack any knowledge or equipment in teaching the online course?
- What do you think are the disadvantages and advantages of online teaching, and what difficulties (if any) did you encounter in teaching the online course?

**Table 2:** Lecturer questionnaires: Views regarding online teaching: Research variables, example statements, and Cronbach’s alpha values.

Research variable	No. of statements	Scale	Statement example	Cronbach’s alpha value (in the post- questionnaire)
Perceived self-efficacy in online teaching#	2	agree/neutral/disagree	I am able to assess my students’ understanding using technological tools	0.76
Satisfaction with online teaching	3	agree/disagree	I am pleased about switching to online teaching	0.63
Belief that technology promotes teaching#	3	agree/neutral/disagree	Technology helps me teach the course in a way that is clearer to the students	0.81
Technology promotes interaction	4	agree/disagree	By using technology I can facilitate student participation and cooperation	0.640
Student participation and engagement*	2	agree/disagree	In online teaching students ask fewer questions (a contrary statement)	0.73

* Category added to the post questionnaire. # Components of belief in self-efficacy for online teaching.

The three parts of the questionnaire provide data that are of a different nature (quantitative and qualitative) but are complementary. Whereas the quantitative part enabled us to look for correlations between the variables, the qualitative data explained the revealed phenomena [46].

### Data analysis

**Fig 1:**
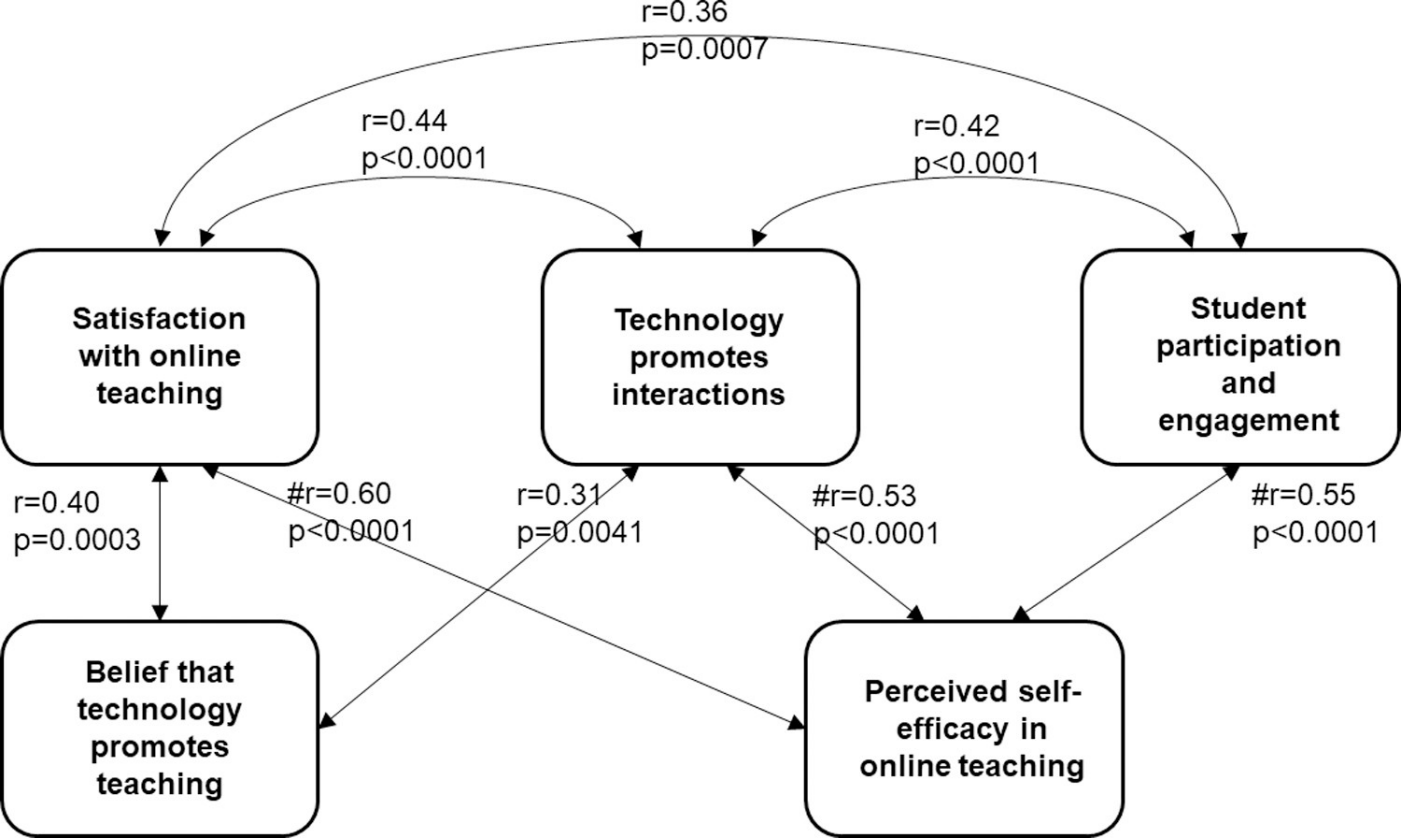
Strong and moderate correlations between the research variables. # High strength of relationship: r value is above 0.5.

Since the data were not normally distributed, we used the non-parametric test approach. The Wilcoxon two-sample test for non-paired pre-and post-comparisons was applied to analyze the differences between the pre-post-questionnaire. Then, we conducted a Spearman correlation test paired to examine correlations between the research variables. We based on Xiao et al. [47] to evaluate the strength of the relationship between the variables: The high strength of relationship: r value is above 0.5; Moderate strength of relationship: r value is above 0.3 and less than 0.5; We did not include in Fig 1 weak correlation–below to 0.3.

To analyze the qualitative data, we used an inductive approach based on the TPACK theoretical framework [48]. Similar to Graham [49]. First, we checked the lecturers’ answers to the open questions against the TPACK knowledge components described in the Introduction. Next, we identified additional components that were impossible to categorize as one of the TPACK knowledge components. These components were found by using grounded theory [50, 51]. One of the authors performed the qualitative analysis, which the three researchers later discussed to resolve any discrepancies. We achieved an entire agreement after a discussion. The same methodology was applied in the qualitative analysis of the advantages and disadvantages of online teaching reported by the participants.

## Results

Here we present separately the research findings obtained by analyzing different parts of the questionnaire. We will begin with the quantitative analysis findings, followed by those of the qualitative analysis.

### Results of the quantitative analysis

The questionnaire’s multiple-choice questions underwent statistical analysis to assess the lecturers’ views about different variables related to online teaching—a Wilcoxon two-sample test of all five variables (see S1 Table and the descriptive statistics presented in S2 Table, in the supplementary materials). Regarding the lecturers’ views in the pre-and post-questionnaires, we could not identify any statistically significant differences between the addressed variables. However, in calculating the correlations between the quantitative variables, we found a different trend in the pre-and post-questionnaires. Only one significant correlation (moderate) emerged in the pre-questionnaire (S3 Table) between the lecturers’ perceived self-efficacy and technology promotes interactions (r = 0.360, p = <0.0001). However, in the post-questionnaire, positive and significant Spearman correlations emerged between the variables, as shown in S4 Table and Fig 1.

Different variables were found to be high/moderate correlated with the two components of self-efficacy. The correlation between these two components was examined, and a low correlation was found. The first component of self-efficacy, “Perceived self-efficacy in online teaching,” was highly significantly correlated with “Satisfaction with online teaching” (r = 0.60, p<0.0001), “Technology promotes interactions” (r = 0.53, p<0.0001), and “Student participation and engagement” (r = 0.55, p<0.0001). The second component of self-efficacy, the variable “Belief that technology promotes teaching” was significantly (moderately) correlated with “Satisfaction with online teaching” (r = 0.40, p = 0.0003) and “Technology promotes interactions” (r = 0.31, p = 0.0041).

Significant Spearman correlations (S4 Table) that were found only in the post-questionnaire might indicate variables related to the development of self-efficacy. Fig 1 summarizes the quantitative findings and shows the moderate and high correlations between variables that were found.

### Results of the qualitative analysis

#### The knowledge lecturers used for online teaching

**Fig 2:**
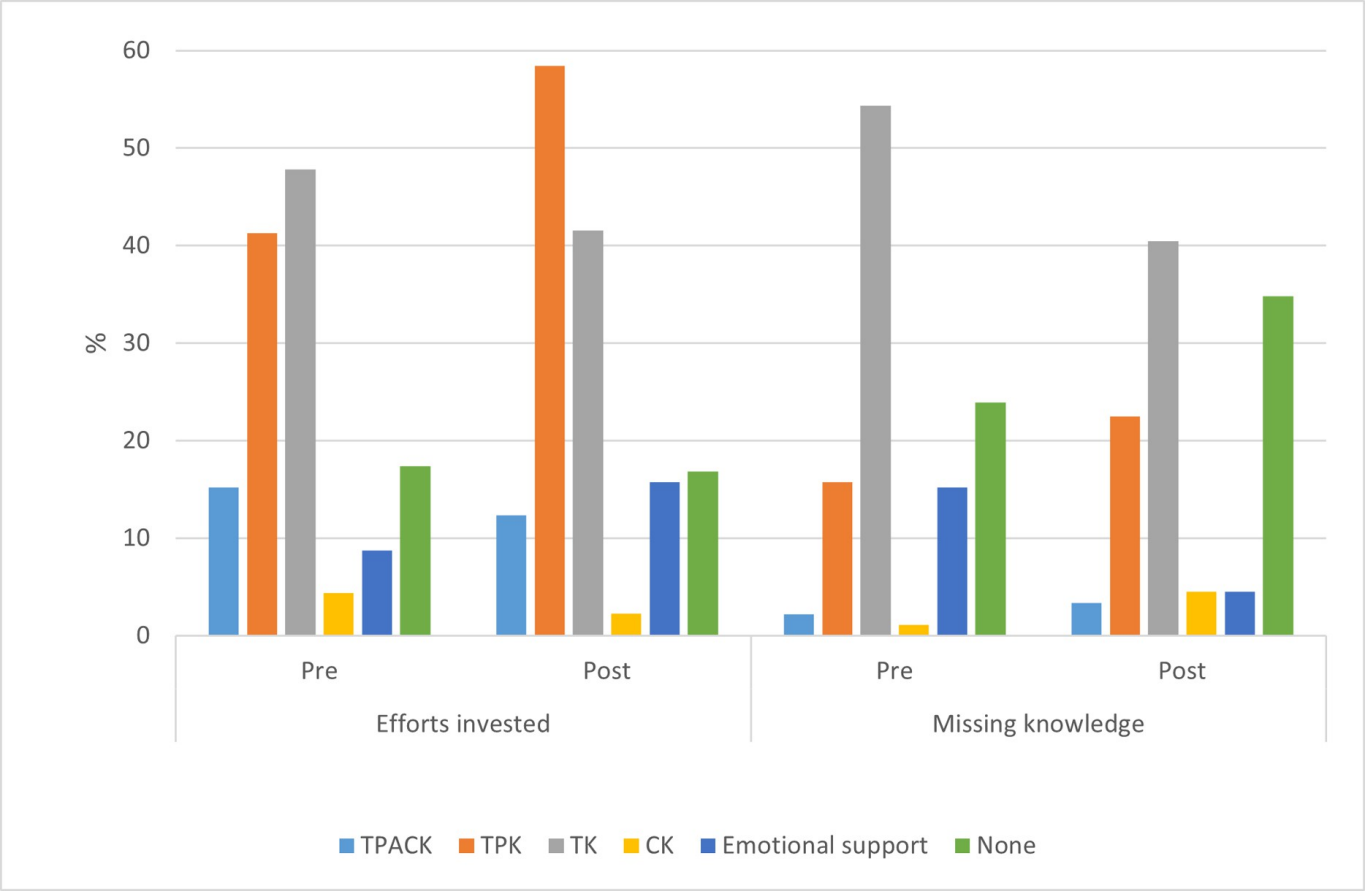
"Missing knowledge" and "Effort invested" in online teaching before and at the end of the semester. Both "Missing knowledge" and "Effort invested" were characterized by TPACK components. The number of participants was used to calculate the percentages for each question.

In the questionnaire completed before online teaching began, the lecturers answered two questions regarding their preparation for online teaching: (1) What efforts did they make in preparing and teaching the online course and (2) what knowledge and equipment did they lack in order to complete the task. These two questions reappeared in the post-questionnaire. Namely, both questions were aimed at determining the current knowledge they used in preparing for remote teaching (question 1) and the knowledge they still need to acquire in order to teach online. Table 3 and Fig 2 present the knowledge components according to the TPACK framework.

**Table 3:** Lecturers’ efforts in preparing an online course before and after experiencing online teaching, categorized by TPACK components.

Category	Category description	Example statements
TK	How to use various features of technological applications (e.g., Zoom)	I tried to understand how Zoom works, and made sure that my PowerPoint slides are seen clearly (pre) I intend to give the students a link to upload their homework to the box, and check it digitally (pre) I did not make significant changes to the materials (presentations, assignments). I invested time in learning the ZOOM options (post).
TPK	Pedagogical aspects of online teaching of a course, mostly on ZOOM: group work, evaluation of understanding, discussion, engaging students in learning	I prepare extra home assignments and cut down the presentations (pre) I added questions that I asked the students during the lecture to make sure they were still present (post) I added reviews to increase student engagement (post)
TPACK	Integration of technology supporting the teaching of a certain (scientific) content: video clips or animation to explain a phenomenon or an experiment, questions to diagnose misconceptions	Changed teaching practices, for example difficulty in explaining, or developing a new formula, require using new media to make knowledge accessible, such as explanatory clips, demonstrations, photographed experiments, and simulations (pre) 1. I gave shorter lectures. 2. I posted the presentation for the students one week before the lecture. 3. At the end of each presentation, I added specific questions corresponding to the difficulties I have come across. (post) We uploaded extended materials and explanations with the lectures, including links to online videos and training. (post) The course focused on data analysis and not on the experiment itself (as it would in a normal year). This enabled me to improve the students’ theoretical skills towards better understanding of the experimental methods. I included real experiment results on screen and was able to provide challenging examples of data analysis. (post)
CK	Critical selection of course contents	When I realized that I would not be able to teach the same amount of material as in a normal year, I went over the course content leaving only the central parts. (post) I skipped all the laboratory experiments and left only the lectures. (post)
None	No effort was made to change the teaching in the course	So far, no effort. (pre). I gave the lectures exactly in the same way as I would frontally (post)
Emotional support	Emotional assistance to students	I was available to students more often and for longer times than in a regular semester.

As Fig 2 shows, the lecturers reported that their preparations for the online course differed from what they had planned prior to teaching online. Forty-eight percent of the lecturers reported about preparations concerning the category “technological knowledge (TK),” i.e., learning how to operate technological tools for education. A smaller portion of the lecturers (42%) reported in the post-questionnaire that they had made great efforts to learn TK in order to teach online. In the techno-pedagogical category (TPK), the lecturers reported that they made considerable efforts in the online implementation of various pedagogies (41% in the pre- and 58% in the post-questionnaire). About 17% of the pre-questionnaire respondents and of the post-questionnaire respondents said they made no effort to adjust the course to the online environment. Notably, most of those lecturers taught in the Faculty of Life Sciences and used presentations. We also noted that the lecturers made increased efforts to address the emotional needs of their students. However, this aspect was mentioned by only 9% of the lecturers in the pre-questionnaires and by 17% in the post-questionnaire.

### Knowledge that the lecturers lacked for online teaching

As part of their preparations for online teaching, lecturers had to indicate what additional knowledge or equipment they needed for online education. Their answers related to knowledge were categorized according to the TPACK components. We analyzed the answers regarding equipment and provided the Weizmann Institute with information regarding what equipment should be purchased for the lecturers. Before online teaching began, equipment was a serious issue. Eighteen of the 46 lecturers complained that they lacked the equipment required for distance teaching. Although several teachers still lacked appropriate equipment at the end of the semester, their number decreased (15 of 89). To address this problem, the Institute purchased online teaching equipment for lecturers during the 2021 academic year. In addition, 15 classrooms are currently being modified for hybrid teaching with online teaching equipment. The classes will mainly serve lecturers who use a whiteboard.

The number of lecturers who stated that they needed no additional knowledge was increased in the post-questionnaire (from 24% to 35%). In contrast to the decreased need for equipment noted at the end of the online teaching semester, the lecturers’ need for various technological knowledge related to pedagogy (TPAK, TPK) increased after they had experienced online teaching (Fig 2). Only three lecturers mentioned the need for comprehensive knowledge about TPACK before the semester began, four lecturers indicated that they needed it at the end of the semester. Such knowledge included, for example, dedicated teaching applications in mathematics and technology, allowing students to perform lab experiments remotely [52]. The need for additional TPK increased after the lecturers had taught during the online semester (from 16% to 23%), since the lecturers had invested much effort to develop their TPK during the semester, much more than they had expected (from 41% to 58%).

### Advantages and disadvantages of online teaching

**Fig 3:**
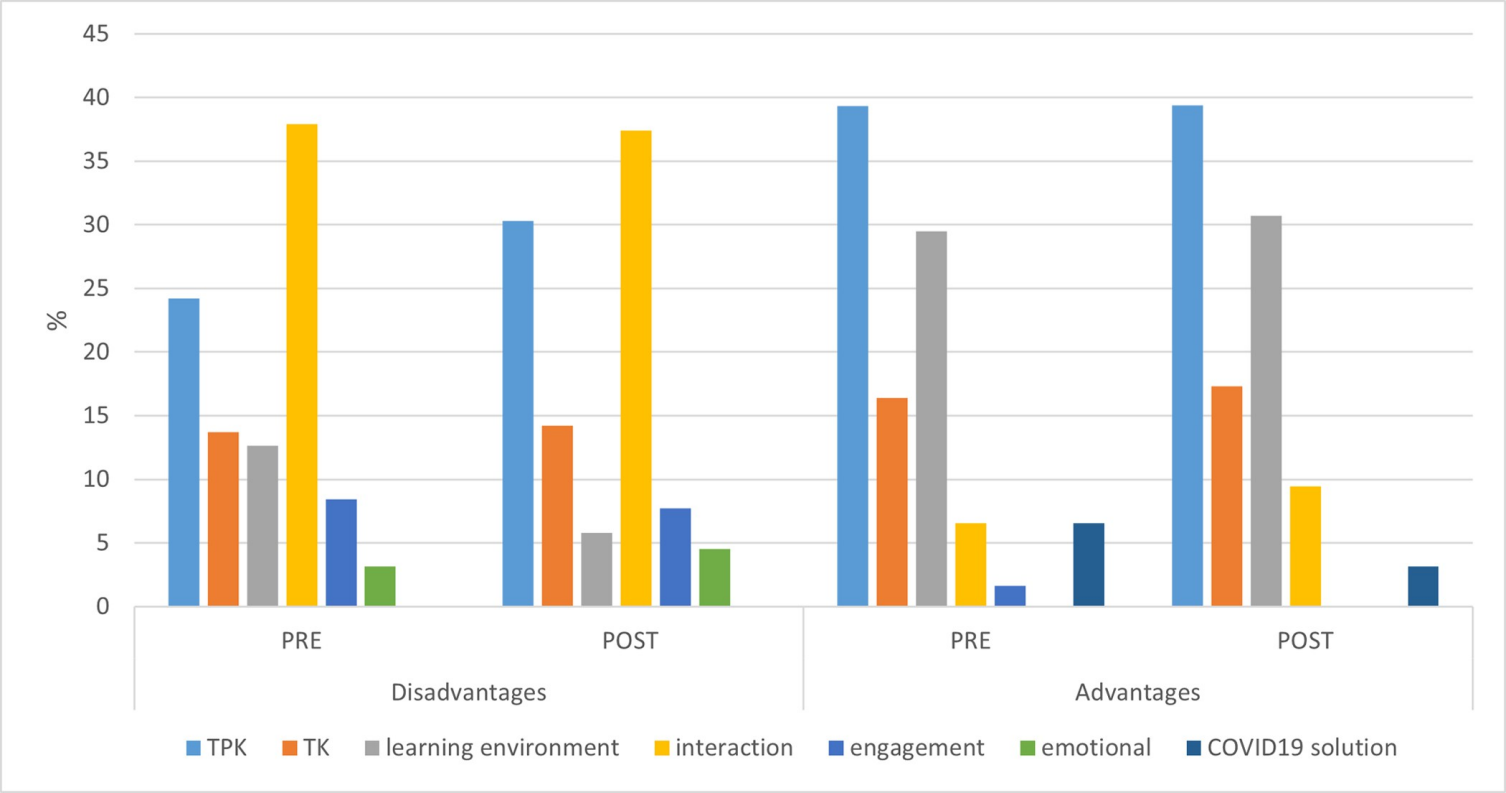
Trends in lecturers’ positive and negative aspects of online teaching before and after utilizing technology for online teaching during the COVID-19 crisis.

The lecturers were requested to provide their input regarding their experience and the challenges they had to cope with during online teaching. They cited both the advantages and disadvantages of their experience. We then categorized their attitudes to reflect different aspects of their experience (see Table 4). The lecturers often reported certain aspects as both advantages and disadvantages of online teaching, as explained in the following and presented in Fig 3. In the pre-questionnaire, only four lecturers (9%) found no advantages in online teaching. Forty-two respondents (92%) mentioned that online teaching had advantages and made 61 statements about its teaching advantages. Eighty-nine lecturers completed the post-questionnaire. Fourteen of them (16%) found no advantages in online teaching; 75 respondents (84%) thought that online teaching had advantages, and made 127 statements regarding the advantages, divided into categories.

**Table 4:** Advantages and disadvantages of online teaching, categorized by TPACK components.

Category	Category description	Examples of advantages of online teaching	Examples of the disadvantages of online teaching
TK	Technological knowledge aspects, the use of various technological applications	Ability to use more online tools (pre) familiar with technological tools and possibilities that are now required but, in the future, we will be able to integrate them into the existing classroom teaching (pre)	There is also a technical disadvantage of writing on an iPad compared to writing on a large blackboard: the field of view is smaller, so the students can only see what the instructor wrote recently, and cannot scroll up to recall what was written earlier (post)
TPK	Techno-pedagogical knowledge. Pedagogical aspects of online teaching of a course, mostly on ZOOM: evaluation of understanding and discussion	Remote online teaching offers a larger variety of strategies for teachers regarding their lesson plans, as well as their assessment methods (post)	Problem assessing the level of understanding in the class. (pre) As a lab course, we missed the hands-on part and we presented only the theoretical part (post)
Learning environment	The setting where the learning occurs	The flexibility of working from different locations (for the students and myself) (post)	Some students/teachers will have a hard time creating a good teaching/learning environment at home—due to kids/pets/others (pre)
Interaction	Lecturer-student contact, remote communication, and the absence of body language and feedback	There was a feeling that the students cooperated and helped each other through the WhatsApp groups which they opened to organize themselves before and during the online learning (post)	The quality of interaction may be less spontaneous and less satisfactory. (pre) They (the students) will not be able to approach us directly during breaks or after the lesson for short informal conversations or questions (pre)
Engagement	Making the students show up to the lesson, get involved, and participate in learning with a positive attitude	Maybe some students will find such additional technical tools appealing (pre)	Not sure how much they will be engaged in the course in this format (pre) Difficulties in gaining the student’s trust and attention and getting them involved and dedicated (pre)
Emotional	Emotional aspects of distance learning and assistance to students	-	A feeling of overload is created, in my opinion, because of the sense of urgency and the excessive sitting. (post)
COVID19 solution	Distance teaching as a learning continuity	we can transfer all the relevant knowledge to our students in a convenient way despite the COVID-19 situation (pre)	-

In contrast to the finding that only some lecturers found no advantages in online teaching, all the lecturers mentioned negative aspects regarding online teaching (95 statements in the pre questionnaire, and 155 statements in the post-questionnaire).

Some of these aspects in the pre questionnaire concerned only the lecturer, others only the students, but most were concerned both. The main negative factor in distance learning was the difficulty maintaining lecturer-student interaction, mentioned by 37% of the lecturers. This disadvantage had several aspects: difficulty maintaining lecturer-student contact, lack of the students’ physical presence, lack of group work and peer learning, difficulty in remote communication, and the absence of body language and feedback, making communication even more difficult. Another significant negative aspect was the difficulty that the lecturers had with techno-pedagogical knowledge (TPK). They were used to applying a specific pedagogy in face-to-face teaching and mentioned that under the new circumstances, they would not be able to conduct discussions and respond to questions during the lectures.

However, after having experienced online teaching over the semester, the lecturers indicated that the number of disadvantages mentioned had increased. Again, the main negative factor concerned the interaction between lecturers and students. However, in the post-questionnaire, the lecturers mentioned negative factors categorized as techno-pedagogical knowledge (TPK). They indicated that the changed teaching mode and the difficulty in evaluating students’ understanding hindered distance teaching and learning. The difficult interaction with the students may also constitute an emotional disadvantage. Interestingly, the number of lecturers who found no advantage in online teaching increased in the post-questionnaire. After having experienced online teaching, about 16% of the post-questionnaire respondents indicated that they found no advantage in distance learning.

The main aspect mentioned in both the pre-and post-questionnaires as promoting distance teaching and learning concerned the learning environment and included techno-pedagogical advantages related to students. Quite a few lecturers noted that distance learning was more convenient since it was not location-dependent, it was flexible in terms of time and did not require commuting. From the techno-pedagogical perspective, the online learning materials and asynchronous learning provided students with a different learning experience and served as significant promoters of learning.

A positive aspect of distance learning that emerged as particularly relevant to the Covid-19 crisis concerned learning continuity. In both questionnaires, the lecturers indicated that the mere existence of the distance teaching option was a significant advantage in itself; had it not been for this technology, none of the courses would have been taught at all.

## Discussion

In the discussion, we provide answers to the research questions and discuss the results.

*1) What variables contribute to developing lecturers’ perceived self-efficacy in online teaching?*

In online teaching, self-efficacy beliefs consist of two aspects: (1) self-efficacy in teaching in an online environment and (2) the belief that technology promotes teaching and learning [34, 35, 53]. The present study measured these aspects before and after a whole semester of online teaching. No significant difference in lecturers’ self-efficacy in online teaching emerged between the administration of the pre-and post-questionnaires. However, the literature reports that experience helps develop a sense of self-efficacy [54]. A possible explanation for this may be that developing self-efficacy in teaching, specifically in online teaching, is a process that occurs over a long time [30, 40], whereas in the present case, the lecturers had to develop online teaching skills over a single semester. Namely, the one-semester Covid-19 teaching experience was insufficient to influence their self-efficacy beliefs. Another possible explanation is that to develop a sense of self-efficacy, one must consider an experience as favorable, as is discussed next. This results are similar to what was found in a study that investigated instructional changes of higher education lecturers from 201 institutions regarding online teaching and learning [55], that have found only small changes in beliefs about online teaching.

As shown in Fig 1, the lecturers’ satisfaction with online teaching, their views about students’ participation and engagement, and their perception that technology promotes interactions are highly and significantly correlated with their perceived self-efficacy in implementing technology in teaching. In addition, the lecturers’ satisfaction with online teaching and their perception that technology can promote interactions are significantly (but moderately) correlated with the lecturers’ belief that technology can promote teaching.

The correlations presented in Fig 1 are derived from the existing psychological literature on self-efficacy. Namely, they consist of two components [53]. Next, we compare these correlations to the findings of the qualitative analysis.

### Satisfaction with online teaching and the belief that technology promotes learning

The correlations presented in Fig 1 indicate that satisfaction with online teaching is correlated with the belief that technology promotes teaching. The qualitative analysis revealed that at the end of the semester, after having experienced online teaching, about 16% of the lecturers did not find any factors that promoted learning in an online environment. They also identified more negative than positive aspects regarding teaching in a technological environment. This means that the lecturers’ satisfaction with online teaching was low. This could lead to a low belief in the ability of technology to promote teaching.

Analysis of the negative aspects mentioned by the lecturers revealed that 37% of them were concerned with the limited interaction between lecturers and students and among the student body. Research literature dealing with online teaching mentions that one important reason why lecturers favor online teaching is their impression that it makes the students more engaged in interactive communication with the lecturers and their co-learners [56, 57]. A connection emerged between the lecturers’ satisfaction and better student performance and a correlation between the lecturers’ and the students’ satisfaction with online courses. Various factors may affect student satisfaction, including lecturer-student interaction, learner-content interaction [58], and self-efficacy in using the internet [59, 60]. Students also mentioned convenience as a reason for their satisfaction with an online course, whereas the lack of interaction had the greatest effect on their dissatisfaction [61]. The higher the self-efficacy in technology-based learning, the higher was the satisfaction with an online course, and vice versa [62].

Under the circumstances of Covid-19, the need arose to switch to online teaching to overcome the emergency measures caused by the pandemic [7]. This switch required speedy learning of online teaching tools and the skills necessary to use them. In this situation, both the lecturers and students faced numerous challenges, making it more challenging to generate and maintain satisfaction than under normal conditions. Weidlich and Kalz (2021) [63] applied the resilience perspective to the experiences of the higher education lecturers. They found that the shift to online teaching and learning at higher education institutes, upon the outbreak of Covid-19, was influenced by the personality traits of the lecturers, as well as their previous experience. These variables supported their resilience and their ability to maintain quality teaching despite the challenges brought about by the pandemic. These results support the current study because self-efficacy beliefs were positively connected to resilience (e.g., [64]).

### The variables “Technology promotes interactions”, “Student participation and engagement”, and “Belief in self-efficacy in online teaching”

The present research findings indicate that personal experience and environmental feedback are correlated with self-efficacy in technology-supported teaching, thus in agreement with the self-efficacy framework [10, 31]. Interaction emerged as an important variable in distance teaching, given the physical distance between the lecturer and learner [65]. As already mentioned, interaction (or the lack of it) emerged as a central disadvantage of the online learning, much like the extent of student engagement. This aspect is essential in the context of online learning since online teaching and learning could reduce student engagement [66]. When such factors inhibit learning, the lecturers have a negative teaching experience that undermines their belief in their ability to teach online. Evidence from similar studies that investigated teachers’ self-efficacy in technology-based teaching and learning supports these findings [31, 35, 67].

*2) Which TPACK components did higher education science lecturers implement in online teaching?*

The current study revealed that the lecturers made significant efforts to acquire varied knowledge upon switching to online teaching. After categorizing the bodies of knowledge they had implemented according to the TPACK framework, we discovered that the main effort concerned learning and implementing TK and TPCK (Fig 2). The trend emerged with regard to TPK, and the number of lecturers who implemented such knowledge in their teaching increased. Whereas the effort concerned TK described. Very few lecturers indicated that they made an effort to integrate technological-pedagogical content (TPACK, Fig 2) both before the semester began and at its end. A similar picture emerged in mapping the specific types of knowledge that the lecturers stated that they had lacked. The analysis revealed that the lecturers had been focusing on learning the technologies required for online teaching (TK), as well as ways to use the emerging technologies as pedagogical tools (TPK) in teaching (in particular, to generate interaction). Very few lecturers described how they used technological tools to enhance the learning of certain scientific content (TPACK) in their courses. Moreover, the lecturers seldom mentioned any need to learn or implement such tools. A full implementation of the TAPCK requires rethinking about the course content and pedagogy in light of the applied technology and was rarely detected in the current study. Similar results were obtained in a study that investigated instructional changes investigated by university faculty during the Covid-19 pandemic [55]. They have applied the Puentedura’s Substitution-Augmentation-Modification-Redefinition (SAMR) model analyzed the level of instructional changes made by faculty [68]. Their analysis reveal that “university educators, on average, converted their existing courses to online courses with some functional improvements (Augmentation) and a modest revision of critical course redesign components (Modification), without quite reaching the level of creating new tasks that were previously inconceivable (Redefinition). This level of change may be regarded as a substantial outcome given that educators were required to create online courses in a short period of time and without much preparation or assistance.” [53, p. 13].

Lecturers at the institutes of higher education are experts in content knowledge (CK) and have experience in lecture-based teaching (PK). However, even those lecturers with many years of teaching experience are novices regarding online teaching. Analysis of the results revealed that the lecturers initially focused on learning how to use the technological tools (TK) in order to be able to teach their course (for example, using Zoom). Discussions have been underway at the Weizmann Institute about the need to learn how to use the learning management system Moodle, which the Institute lecturers do not universally use. Moodle is extensively used worldwide, with over 212 million users (stats.moodle.org). The system’s Hebrew version is in broad use in Israel’s institutes of higher education and at the high-school level [69] since it addresses the needs of both administrators and educators [70]. Given the need that arose during the covid pandemic, the Moodle system was made available for use by all of the Institute’s lecturers starting in the 2021 academic year.

During the semester, the lecturers said they sensed a lack of contact with the students and had little interaction that required learning and implementing the techno-pedagogical (TPK) knowledge. To resolve this problem, the lecturers resorted to using technological tools such as conducting polls or learning in small groups using the “breakout rooms” option in a way to make the students more engaged in the process. They identified the importance of being socially present in an online course, defined as vital in various studies of learning in online environments as was reported in previous studies [71, 72]. They also pointed out the difficulty of engaging students in learning under the extraordinarily difficult conditions of the Covid-19 pandemic. Oliveira et al. (2021) [73], who conducted a study regarding ICT platforms during the pandemic, found that the experience of using the ICT platforms was generally positive. However, both students and lecturers negatively perceived the need for personal adaptations [73]. Under these pandemic conditions, lecturers had to learn to implement different knowledge, which we termed “emotional support” [74]. This difficulty has repeatedly appeared in numerous articles dealing with online teaching under Covid-19 conditions [75]. Although emotional presence occurs spontaneously in frontal teaching, in an online course, it is only possible if the lecturer makes a specific effort. It is a vital component of online learning [74].

As online teaching novices, very few lecturers have time to spare to learn and implement technological tools capable of promoting the teaching of their specific course (i.e., TPACK knowledge). Moreover, they even fail to mention this body of knowledge as lacking. In their learning of online teaching, the Weizmann Institute lecturers began by acquiring the most general technological knowledge (TK). Then, they applied those tools to their pedagogy, namely, using techno-pedagogical knowledge (TPK). Finally, they implemented tools that responded to their students’ socio-emotional state of mind during this crisis period. Interestingly, similar findings were reported before the pandemic [49] and they also emerged in a study conducted during the pandemic in China, which investigated how Chinese universities prepared for distance learning during the Covid-19 crisis [76]. Lecturers in China’s top universities also focused on the technical aspects of transitioning to an online environment rather than on the pedagogy of online teaching of course content. The situation in the UK was similar, where rapid online immigration interfered with the pedagogical performance and the personal lives of lecturers [77]. These findings significantly contribute to the research about TPACK in institutes of higher education.

We assume that the lecturers’ way of transforming their course to an online platform also affected their different learning bodies of knowledge. The Weizmann Institute lecturers had the option of participating in two online sessions given by the first author of this article, which offered guidance in using Zoom for online teaching, as was done in other places around the world [42, 43]. This should be followed by learning and implementing technological tools in content teaching. To this end, we recommend holding online teaching sessions for specialized groups of lecturers teaching the same field of knowledge (for example, mathematics or life sciences). As was shown in different studies during the pandemic that teaching each of the disciplines requires specific understanding of technology that can be applied for teaching (e.g., mathematics teaching during the pandemic [78]. In such sessions, the TPACK body of knowledge would interest all the group members; this could promote learning this complex knowledge and its implementation at the Weizmann Institute.

## Research limitations

The study had limitations regarding the quantitative part of the research. Although we included more than 50% of the lecturers in the second semester, the number of lecturers (46 in the pre, 89 in the post) who filled out the questionnaire was not high. We, therefore, present here a correlation analysis rather than a more advanced statistical analysis. In addition, we used an unequal sample size in the pre-and the post-samples. This is important if certain assumptions are not met, such as homoscedasticity, but these are irrelevant in Wilcoxon tests. Namely, the sample sizes do not have to be similar in this case.

Owing to economic reasons during the pandemic, short scales were applied to the questionnaire’s items. We hope that the results will serve as a basis for further research and advanced statistical analysis.

## Research implication

Pokhrel and Chhetri (2021), who reviewed the challenges and opportunities in higher education during the pandemic, suggest that “The Covid-19 pandemic has provided us with an opportunity to pave the way for introducing digital learning.” [79, p. 133]. In this study, we drew conclusions and learned lessons that can be applied more generally from this pandemic to improve online teaching in higher education institutions. The evaluation of lecturers’ TPACK and self-efficacy can assist in the future design of professional development programs for online teaching in higher education. Specifically, we identified the need for graduate science training programs to integrate the TPACK components. In addition, weidentified the need to advance the professional development of lecturers in online teaching, specifically, the need to support the development of self-efficacy for online teaching in higher education.

Pajares (1992) argued that knowledge and belief are intertwined [80]. This is supported by a number of past studies that found a link between TPACK and a sense of self-efficacy among teachers and emphasized the importance of the teachers’ feelings about the integration of technologies. Joo et al, for example, showed that TPACK may influence the self-efficacy beliefs of teachers and indicated that this consequently affects the integration of technology [81]. Another study pointed to TPACK as predicting the sense of self-efficacy for integrating technology in teaching among preservice teachers [82]. However, in this research our focus was on the individual contribution of each theory (TPACK and self-efficacy) to the overall teaching experience. Future research could examine their mutual effect and examine, for example, whether acquiring TPACK correlates with the level of self-efficacy.

Nowadays, the need for such professional development programs for higher education lecturers is stronger than ever. Although some countries are experiencing some sort of return to normal, most higher education institutions are still shifting back and forth between in-person and online teaching or integrating some kind of hybrid model. Furthermore, going forward, many academic institutions are considering integrating online and hybrid education as their default model. This reality stresses the need to rethink the existing teaching methods as well as the growing importance of introducing online teaching methods in professional development training. Our findings can contribute to the development of more efficient professional development and training regarding online teaching.

## Conclusion

Here, we identified the specific knowledge and skills required to teach in institutes of science higher education under the current difficult circumstances of Covid-19, and we examined factors that might help to develop self-efficacy in technology-based instruction. Note that these findings reflect the situation during an ongoing emergency but could be applicable to online instruction in general [7]. In future research of online teaching in higher education institutes, one should distinguish between the general characteristics of online teaching and specific phenomena that emerged during this time, as a result of the pandemic, for example. Lecturers should receive guidance to facilitate their gradual learning of knowledge and skills required for emergency online teaching. Special attention should be given to identify the best way to obtain this knowledge in a way that will include attention to lecturers’ attitudes and that will support the development of self-efficacy for online teaching, since knowledge and self-efficacy together can improve online teaching, and both are vital both under routine circumstances and during an emergency.

## Supporting information

S1 Table Wilcoxon two-sample test for non-paired pre-and post-comparisons. (PDF)

S2 Table Descriptive statistics of quantitative research variables means and standard deviation. (PDF)

S3 Table Correlation (Pearson test) between quantitative variables in the pre-questionnaire. (PDF)

S4 Table Correlation (Pearson test) between quantitative variables in the post-questionnaire. (PDF)

S5 Table Pre-post questionnaire data. (XLSX)
