# Correction: What can be learned from lecturers’ knowledge and self-efficacy for online teaching during the Covid-19 pandemic to promote online teaching in higher education

**DOI:** 10.1371/journal.pone.0314398

**Published:** 2024-11-21

**Authors:** The PLOS ONE Staff

The ORCID iD is missing for the first author. Please see the author’s respective ORCID iD here:

- Author Ron Blonder’s ORCID iD is: 0000-0003-4796-4678 (https://orcid.org/0000-0003-4796-4678).

The publisher apologizes for the error.
