# Supplementary material for: What can be learned from lecturers’ knowledge and self-efficacy for online teaching during the Covid-19 pandemic to promote online teaching in higher education
Source: PLoS One. 2022 Oct 5;17(10):e0275459. doi: 10.1371/journal.pone.0275459 (PMC9534420; doi:10.1371/journal.pone.0275459)
Supplement: S1 Table — (PDF) [file pone.0275459.s001.pdf]

Table S1. Wilcoxon two-sample test for non-paired pre-and post-comparisons

	Pre-Post
Perceived self-efficacy in online teaching	(z=1.5325, p=0.0627)
Satisfaction with online teaching	(z=1.4095, p=0.0793)
Belief that technology promotes teaching	(z=1.1451, p=0.126)
Technology promotes interactions	(z=0.4647, p=0.3211)
