# Supplementary material for: What can be learned from lecturers’ knowledge and self-efficacy for online teaching during the Covid-19 pandemic to promote online teaching in higher education
Source: PLoS One. 2022 Oct 5;17(10):e0275459. doi: 10.1371/journal.pone.0275459 (PMC9534420; doi:10.1371/journal.pone.0275459)
Supplement: S2 Table — (PDF) [file pone.0275459.s002.pdf]

Table S2. Descriptive statistics of quantitative research variables means and standard deviation

	Means		STD. Deviation	
	Pre	Post	Pre	Post
Perceived self-efficacy in online teaching#	0.44	0.50	0.29	0.36
Satisfaction with online teaching	0.60	0.63	0.22	0.36
Belief that technology promotes teaching#	0.20	0.21	0.24	0.37
Technology promotes interactions	0.17	0.24	0.20	0.32
Student participation and engagement	NA	0.40	NA	0.44
