# Supplementary material for: What can be learned from lecturers’ knowledge and self-efficacy for online teaching during the Covid-19 pandemic to promote online teaching in higher education
Source: PLoS One. 2022 Oct 5;17(10):e0275459. doi: 10.1371/journal.pone.0275459 (PMC9534420; doi:10.1371/journal.pone.0275459)
Supplement: S3 Table — (PDF) [file pone.0275459.s003.pdf]

Table S3. Correlation (Pearson test) between quantitative variables in the pre- questionnaire.

N=46. # – components that build up lecturers' self-efficacy belief in their ability to teach online

	Perceived self-efficacy in online teaching#	Satisfaction with online teaching	Belief that technology promotes teaching#	Technology promotes interactions
Perceived self-efficacy in online teaching#	1.00000	0.231 (0.122)	0.182 (0.225)	0.360 (<.0001)
Satisfaction with online teaching		1.00000	0.213 (0.154)	0.072 (0.633)
Belief that technology promotes teaching#			1.00000	0.019 (0.895)
Technology promotes interactions				1.00000

We used Xiao et al., 2016 to evaluate the strength of the relationship between the variables:

High strength of relationship: r value is above 0.5

Moderate strength of relationship: r value is above 0.3 and less than 5

We did not include in Figure 2 weak correlation – below to 0.3
