# Supplementary material for: What can be learned from lecturers’ knowledge and self-efficacy for online teaching during the Covid-19 pandemic to promote online teaching in higher education
Source: PLoS One. 2022 Oct 5;17(10):e0275459. doi: 10.1371/journal.pone.0275459 (PMC9534420; doi:10.1371/journal.pone.0275459)
Supplement: S4 Table — (PDF) [file pone.0275459.s004.pdf]

Table S4. Correlation (Pearson test) between quantitative variables in the post- questionnaire. N=89. # – components that build up lecturers' self-efficacy belief in their ability to teach online

	Perceived self-efficacy in online teaching#	Satisfaction with online teaching	Belief that technology promotes teaching#	Technology promotes interactions	Student participation and engagement
Perceived self-efficacy in online teaching#	1.00000	0.60 (<.0001)	0.30 (0.0064)	0.53 (<.0001)	0.55 (<.0001)
Satisfaction with online teaching		1.00000	0.40 (0.0003)	0.44 (<.0001)	0.36 (0.0007)
Belief that technology promotes teaching#			1.00000	0.31 (0.0041)	0.06 (0.5635)
Technology promotes interactions				1.00000	0.42 (<.0001)
Student participation and engagement					1.00000

We used Xiao et al., 2016 to evaluate the strength of the relationship between the variables:

High strength of relationship: r value is above 0.5

Moderate strength of relationship: r value is above 0.3 and less than 5

We did not include in Figure 2 weak correlation – below to 0.3
